# Study on predictive factors of blood culture results in leukemia patients with fever during chemotherapy

**DOI:** 10.1097/MD.0000000000032576

**Published:** 2023-01-13

**Authors:** Mengjia Liu, Ying Wu, Peijing Qi, Ruidong Zhang

**Affiliations:** a Hematology Oncology Center, Beijing Key Laboratory of Pediatric Hematology and Oncology; Key Laboratory of Major Diseases in Children, Ministry of Education; National Key Discipline of Pediatrics; Beijing Children’s Hospital, Capital Medical University, Beijing, China.

**Keywords:** blood culture, infection, leukemia, prediction model

## Abstract

Infection is a common complication of leukemia patients undergoing chemotherapy. Blood culture results are often needed to guide clinical use, but repeated sampling is often necessary to improve the positive rate and eliminate contamination. The purpose of this paper is to find predictive factors of blood culture results among clinical and laboratory indicators and try to establish a prediction model, so as to better choose the time of blood culture examination, predict the results, and better guide clinical treatment. We retrospectively collected clinical and laboratory data of febrile acute leukemia patients undergoing chemotherapy. The samples were randomly assigned to the training set and the validation set, and the prediction model was constructed from the training set. The calibration curve was made in the validation set and the Hosmer-Lemeshow test was performed to evaluate the prediction performance of the prediction model. A total of 229 patients were included. Univariate and multivariate analyses suggested that temperature at fever and procalcitonin were variables of significant difference between positive and negative blood culture patients. The sensitivity of the 2 variables for predicting blood culture results was high, but the specificity was low. In the process of external validation, the predictive ability of the constructed prediction model to the blood culture results was low. This study identified clinical and laboratory parameters associated with blood culture outcomes, but the predictive model established has low predictive accuracy in external validation.

## 1. Introduction

Infection is one of the major complications in patients with leukemia during chemotherapy.^[1]^ Early identification of pathogen can better guide clinical treatment and not only can promote infection control as soon as possible, but also can reduce unnecessary antibiotic use. Blood culture is one of the important tests to obtain pathogen results, but due to the positive rate of blood culture itself and the possibility of contamination,^[2]^ especially in cases where fever is accompanied by neutropenia,^[3]^ it is often necessary to perform a blood culture test prior to the empiric use of broad-spectrum antibiotics and repeat the test later in the course of the disease. Therefore, the recognition of predictors and establishment of a predictive model based on clinical and laboratory indicators to predict the results of blood culture can make the time of blood culture more appropriate, reduce the medical costs of repeated blood culture, reduce the pain of repeated sampling in children, and improve the positivity of blood culture, so as to better guide the clinical anti-infection decision-making, more targeted drug use, which can significantly improve the prognosis of patients.^[4–6]^ There have been many studies of infection-related clinical and laboratory indicators, but few studies have been done to establish predictive models of blood culture outcomes.^[7]^ The purpose of this study was to find predictive factors and establish and validate a predictive model for blood culture results based on clinical features and laboratory parameters.

## 2. Methods

(1). Patient: Our data comes from the medical record management system of inpatients in Beijing Children’s Hospital. Retrospective analysis was done for patients with acute leukemia diagnosed by bone marrow morphology at the Hematology and Oncology Center of Beijing Children’s Hospital from July 2019 to December 2020. The inclusion criteria were as follows: Patients with fever, defined as the temperature measured by axillary temperature > 37.5 degrees Celsius. For patients with multiple blood culture results, we chose the first positive blood culture result; The control group was the patients with fever, but the blood culture results were negative. If there were multiple blood culture results, one of them was randomly selected. Exclusion criteria: Fever considered to be related to other reasons such as drug use; If a variety of pathogens are seen in the culture results, it is generally considered to be contaminated, but if this is not consistent with clinical situation and treatment is based on the blood culture results, it is not considered to be contaminated. Incomplete data records in the record system.
(2). Variables Collected: Basic information about the patient, whether the blood culture was positive, sampling stie of positive blood culture(peripheral blood, peripherally inserted central catheter [PICC] or both), temperature at fever, time to positivity (TTP), When there are multiple positive results, take the shortest time), C reactive protein, complete blood cell analysis, blood biochemistry, coagulation, procalcitonin (PCT), cytokines, the application of antibiotics before blood collection,^[8]^ Nosocomial infection (defined as more than 48 hours after admission and < 48 hours after discharge),^[9]^ type of pathogen. Blood samples should be taken at the time of fever as the preferred choice, otherwise they should be extracted within 24 hours before and after fever.
(3). Statistical analysis: The prediction model establishment did not include indicators with > 20% missing values, and the remaining data were filled using multiple imputation. The “mice” package of R software was used to obtain estimated data sets. We randomly divided all remaining febrile leukemia patients into training (70%) and validation (30%) cohorts. The training cohort was used to construct a prediction model, and the validation cohort was used to perform external validation. Categorical variables were described as frequency and percentage values, and differences between cohorts were determined using the chi-square or Fisher’s exact test. Continuous variables were described as mean and standard-deviation values or median and interquartile-range values depending on whether or not they conformed to a normal distribution.

Independent risk factors for blood culture result in febrile leukemia patients were determined using logistic regression. The determined independent prognostic factors were again analyzed using a logistic regression model, and the results were expressed as odds ratios and 95% confidence intervals (CIs).

Multiple indicators were used to externally validate the nomogram. The area under the receiver operating characteristic curve (AUC) was used to evaluate the recognition ability of the prediction model. We also constructed a calibration curve and conducted a Hosmer-Lemeshow test to evaluate the calibration of the prediction model.

R (version 4.0.3) were used for the statistical analyses, and *P* < .05 was considered statistically significant.

This study has been approved by the local ethics committee of Beijing Children’s hospital.

## 3. Results

### 3.1. Univariate analysis

**Figure 1.**
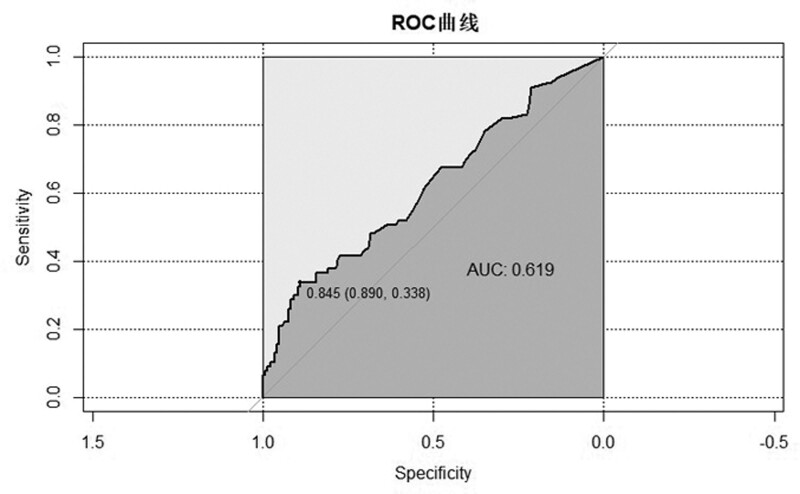
ROC curve and cutoff value of PCT for blood culture results. PCT = procalcitonin, ROC = receiver operating characteristic curve.

**Figure 2.**
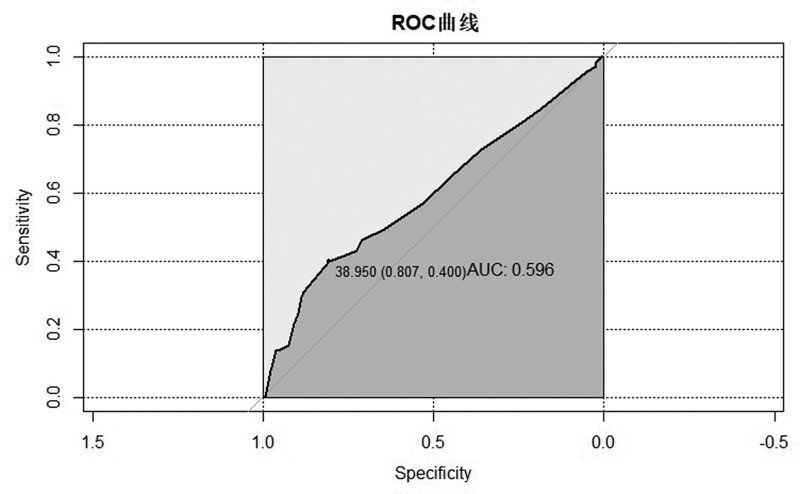
ROC curve and cutoff value of temperature at fever for blood culture results. ROC = receiver operating characteristic curve.

A total of 229 patients were included in the study. A total of 79 patients had positive blood cultures. A comparison of basic information for patients with positive and negative blood culture is summarized in Table 1. There were no significant differences in gender, age, body weight, risk classification, length of hospital stay, whether antibiotics were used before, but significant differences in temperature at fever, PCT. However, although there were statistically significant differences in temperature between the 2 groups, there is only a minor difference in absolute value of temperature at fever (blood culture negative vs blood culture positive 38.59 ± 0.57 vs 38.74 ± 0.65). The receiver operating characteristic curve (ROC) curves and cutoff values of temperature at fever and PCT for blood culture diagnosis are shown in Figure 1 and Figure 2. When the cutoff value of PCT is 0.845 ng/mL, the AUC is 0.619, the sensitivity is 0.89, and the specificity is 0.338. When the cutoff value of temperature at fever is 38.95 ° C, the AUC is 0.596, the sensitivity is 0.807, and the specificity is 0.4.

**Table 1:** Comparison of basic information in patients with positive and negative blood culture.

	Level	Overall	Positive	Negative	*P*
n		229	150	79	
Sex (%)	Male	125 (54.6)	80 (53.3)	45 (57.0)	.7
	Female	104 (45.4)	70 (46.7)	34 (43.0)	
Weight (mean [SD])		21.51 (11.53)	22.00 (12.49)	20.59 (9.46)	.382
Age (mean [SD])		5.99 (4.38)	5.95 (3.89)	6.08 (5.22)	.824
Risk group (%)	SR	68 (29.8)	46 (30.9)	22 (27.8)	.856
	MR	130 (57.0)	83 (55.7)	47 (59.5)	
	HR	30 (13.2)	20 (13.4)	10 (12.7)	
Temperature at fever (mean [SD])		38.59 (0.57)	38.51 (0.51)	38.74 (0.65)	.007
Length of hospital stay (mean [SD])		58.38 (168.19)	65.61 (201.88)	44.66 (67.39)	.371
Application of antibiotics before blood collection (%)	No	179 (78.2)	118 (78.7)	61 (77.2)	.933
	Yes	50 (21.8)	32 (21.3)	18 (22.8)	
White blood cell count (mean [SD])		3.37 (8.07)	2.77 (3.34)	4.50 (12.87)	.124
Neutrophil count (mean [SD])		2.38 (7.28)	1.84 (2.95)	3.46 (11.85)	.122
Platelet count (mean [SD])		139.24 (124.78)	137.11 (105.94)	143.27 (155.07)	.725
HCT (mean [SD])		27.90 (7.16)	28.05 (7.99)	27.61 (5.36)	.659
CRP (mean [SD])		18.44 (27.74)	18.97 (28.43)	17.47 (26.57)	.7
PCT (mean [SD])		1.76 (8.16)	0.60 (1.55)	3.97 (13.51)	.003

CRP = C reactive protein, HR = high risk, MR = medium risk, SR = standard risk, PCT = procalcitonin.

### 3.2. Results of multivariate analysis

**Figure 3.**
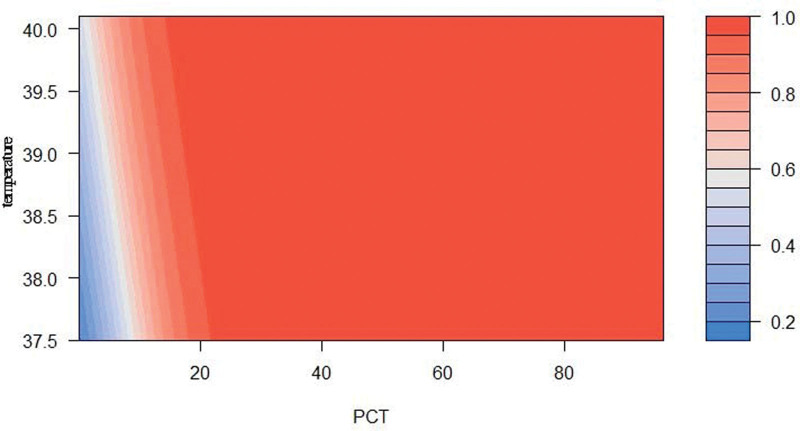
The probability of positive results of blood culture under different PCT and temperature. Different colors represent the probability of positive blood culture, see the legend. PCT = procalcitonin.

Multivariate analysis of blood culture results and the above-mentioned clinical and laboratory indicators in positive and negative blood culture patients still indicated that temperature at fever and PCT were the variables with significant differences between the 2 groups (temperature at fever OR = 1.81, 95% CI was 1.04–3.16, *P* value was .036; PCT OR = 1.22, 95% CI 1.05–1.51, *P* value .03), see Table 2. The probability of positive blood cultures in patients with different PCT and temperature at fever is shown in Figure 3.

**Table 2:** Results of multiple regression.

	Exp (coef) [confint]	*P*
(Intercept)	0.00 [0.00, 0.23]	.037
Sex (女)	1.04 [0.57, 1.87]	.905
Weight	0.98 [0.92, 1.02]	.338
Age	1.06 [0.95, 1.23]	.375
Risk group MR	1.25 [0.64, 2.49]	.515
Risk group HR	0.76 [0.24, 2.24]	.63
Temperature at fever	1.81 [1.04, 3.16]	.036
Length of hospital stay	1.00 [1.00, 1.00]	.625
Application of antibiotics before blood collection (yes)	1.29 [0.59, 2.74]	.517
White blood cell count	1.03 [0.99, 1.10]	.291
Neutrophil count	1.03 [0.98, 1.10]	.31
Platelet count	1.00 [1.00, 1.00]	.818
HCT	0.97 [0.92, 1.01]	.191
CRP	0.99 [0.98, 1.01]	.374
PCT	1.22 [1.05, 1.51]	.03

CRP = C reactive protein, HR = high risk, MR = medium risk, PCT = procalcitonin.

### 3.3. Establishment of predictive model

**Figure 4.**
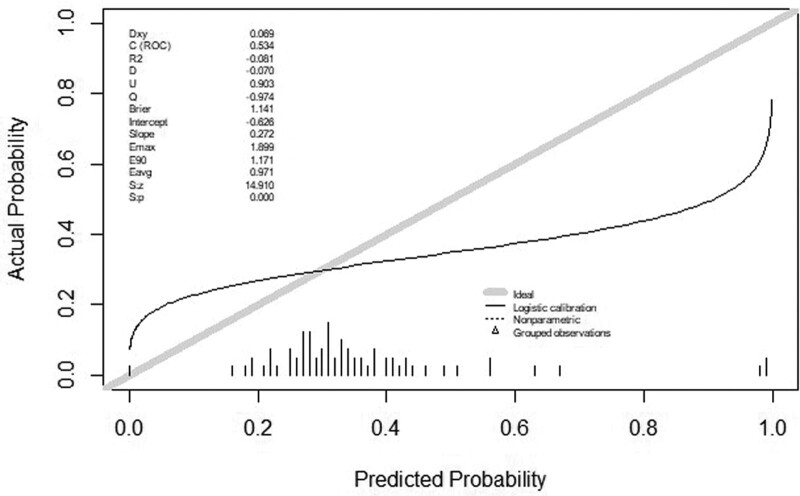
The area under the ROC curve of the calibration curve of the prediction model constructed from the training set is 0.534 in the validation set. ROC = receiver operating characteristic curve.

This article uses Hosmer-Lemeshow Goodness of Fit Test to verify the prediction ability of the model, the *P* value is .4769, the calibration curve of the prediction model constructed by the training set in the validation set is shown in Figure 4, and the area under the ROC curve is 0.693.

### 3.4. Other results

**Figure 5.**
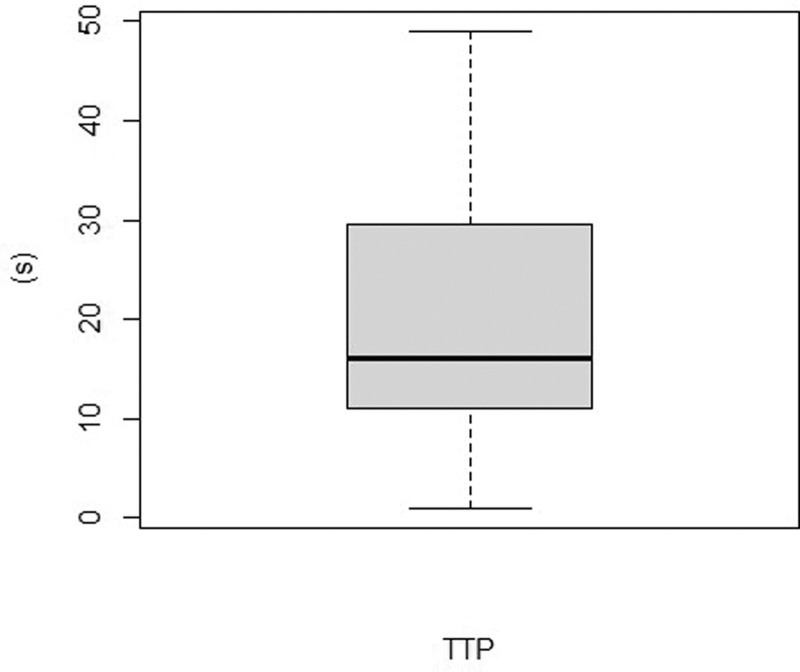
The distribution of TTP (time to positivity) in patients with positive blood culture. TTP = time to positivity.

**Figure 6.**
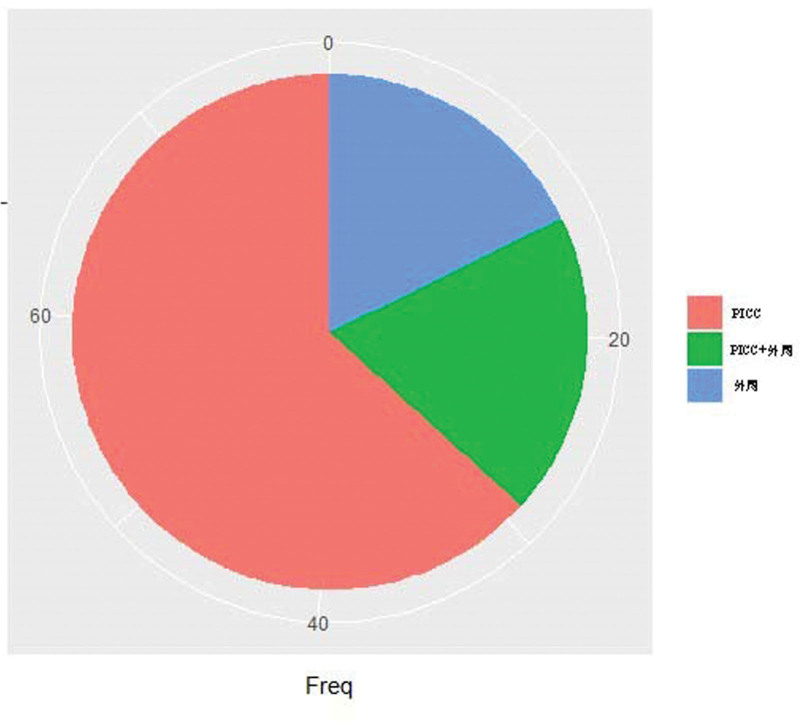
The proportion of sampling site in positive blood culture.

Indicators such as TTP, which represents the time interval between sampling and positive result were also collected. TTP ranges from 1 to 49 hours, with a 25th percentile of 11 hours, a median of 16 hours, and a 75th percentile of 29.5 hours, as shown in Figure 5. At the same time, the proportion of positive blood culture site is shown in Figure 6.

## 4. Discussion

1. Temperature at fever and PCT are independent predictors of blood culture outcome. Among the clinical and laboratory parameters we collected, univariate and multivariate analyses suggested that temperature at fever and PCT are predictive of blood culture results. According to the cutoff value of ROC curve, the sensitivity of the 2 indexes is high, but the specificity is low. PCT is a laboratory indicator that may not be available when deciding whether to perform a blood culture test. Its application is limited in deciding blood culture timing, but it can still be used to predict blood culture results after obtaining blood test results. However, the prediction model we built has a low performance in external validation and cannot build an effective prediction model.
2. The distribution of TTP may suggest the possibility of positive blood culture results at different time periods after sampling.^[10]^ PICC infection is one of the important sources of infection in leukemia patients with chemotherapy fever, and more attention should be paid to the care of PICC.
3. The limitations of this study are as follows: 1. Few of the patients with febrile leukemia in this study had severe clinical manifestations such as severe organ dysfunction and septic shock. Therefore, the results of this study are not applicable to patients with severe infection symptoms and organ dysfunction. 2. This is a retrospective study, some of the clinical information needed to be collected was not recorded in the record management system, and some laboratory tests were not complete in all patients. Prospective collection and recording of patient’s information could reveal more predictive variables.

## 5. Conclusion

Temperature at fever and PCT results are significant predictors of blood culture results, but prediction model established based on them does not perform well in external validation. More clinical and laboratory data collection may be needed.

## Author contributions

**Conceptualization:** Mengjia Liu.

**Data curation:** Ying Wu.

**Formal analysis:** Peijing Qi.

**Resources:** Ruidong Zhang.

## References

[1] Mueller EL Sabbatini A Gebremariam A. Why pediatric patients with cancer visit the emergency department: United States, 2006-2010. Pediatr Blood Cancer. 2015;62:490–5. 25345994 10.1002/pbc.25288 PMC4304987

[2] Paolucci M Landini Mp Sambri V. Conventional and molecular techniques for the early diagnosis of bacteraemia. Int J Antimicrob Agents. 2010;36(Suppl 2):S6–16. 21129933 10.1016/j.ijantimicag.2010.11.010

[3] Lehrnbecher T Robinson P Fisher B. Guideline for the management of fever and neutropenia in children with cancer and hematopoietic stem-cell transplantation recipients: 2017 update. J Clin Oncol. 2017;35:2082–94. 28459614 10.1200/JCO.2016.71.7017

[4] Teh BW Brown C Joyce T. Safety and cost benefit of an ambulatory program for patients with low-risk neutropenic fever at an Australian centre. Support Care Cancer. 2018;26:997–1003. 29018966 10.1007/s00520-017-3921-3

[5] Orme LM Babl FE Barnes C. Outpatient versus inpatient IV antibiotic management for pediatric oncology patients with low risk febrile neutropenia: a randomised trial. Pediatr Blood Cancer. 2014;61:1427–33. 24604835 10.1002/pbc.25012

[6] Haeusler GA-O. Home-based care of low-risk febrile neutropenia in children-an implementation study in a tertiary paediatric hospital. 10.1007/s00520-020-05654-z 32740894

[7] Haeusler GM Sung L Ammann RA. Management of fever and neutropenia in paediatric cancer patients: room for improvement? Curr Opin Infect Dis. 2015;28:532–8. 26381997 10.1097/QCO.0000000000000208

[8] Scheer CS Fuchs C Gründling M. Impact of antibiotic administration on blood culture positivity at the beginning of sepsis: a prospective clinical cohort study. Clin Microbiol Infect. 2019;25:326–31. 29879482 10.1016/j.cmi.2018.05.016

[9] Hess OA-O. The learning hospital: from theory to practice in a hospital infection prevention program. 10.1017/ice.2019.318 31753056

[10] Gavronski S Nogueira KDS. Time to positivity: a useful parameter to evaluate intensive care unit blood stream infections? Rev Bras Ter Intensiva. 2020;32:326–9. 32667441 10.5935/0103-507X.20200049 PMC7405736

